# Causes, effects and connectivity changes in MS-related cognitive decline

**DOI:** 10.1590/S1980-57642016DN10100002

**Published:** 2016

**Authors:** Carolina de Medeiros Rimkus, Martijn D. Steenwijk, Frederik Barkhof

**Affiliations:** 1,2 Department of Radiology, Laboratory of Medical Investigation (LIM-44), Faculty of Medicine of the University of São Paulo, São Paulo SP, Brazil and Department of Radiology and Nuclear Medicine, Neuroscience Campus Amsterdam, VU University Medical Center, Amsterdam, The Netherlands; 1,3 Department of Radiology and Nuclear Medicine, Neuroscience Campus Amsterdam, VU University Medical Center, Amsterdam, The Netherlands and Department of Physics and Medical technology, Neuroscience campus Amsterdam, VU University Medical Center, Amsterdam, The Netherlands

**Keywords:** multiple sclerosis, cognition, brain mapping, functional neuroimaging, diffusion tensor imaging, esclerose múltipla, cognição, mapeamento encefálico, neuroimagem funcional, imagem por tensor de difusão

## Abstract

Cognitive decline is a frequent but undervalued aspect of multiple sclerosis (MS). Currently, it remains unclear what the strongest determinants of cognitive dysfunction are, with grey matter damage most directly related to cognitive impairment. Multi-parametric studies seem to indicate that individual factors of MS-pathology are highly interdependent causes of grey matter atrophy and permanent brain damage. They are associated with intermediate functional effects (e.g. in functional MRI) representing a balance between disconnection and (mal) adaptive connectivity changes. Therefore, a more comprehensive MRI approach is warranted, aiming to link structural changes with functional brain organization. To better understand the disconnection syndromes and cognitive decline in MS, this paper reviews the associations between MRI metrics and cognitive performance, by discussing the interactions between multiple facets of MS pathology as determinants of brain damage and how they affect network efficiency.

## RESUMO

Declínio cognitivo é uma situação frequente mas ainda pouco compreendida na esclerose múltipla (EM). Atualmente, não são totalmente conhecidos os principais determinantes da disfunção cognitiva na doença, tendo sido apontadas fortes associações entre danos à substância cinzenta e declínio cognitivo. Estudos multiparamétricos mostram que os diferentes fatores patológicos da EM participam como causas interdependentes de atrofia da substância cinzenta e dano cerebral permanente. Eles são associados a efeitos funcionais intermediários (detectados por RM funcional) representando um equilíbrio entre desconexão cerebral e alterações (mal) adaptativas. Portanto, uma abordagem de imagem mais abrangente é necessária, com o objetivo de encontrar associações entre alterações estruturais e a organização funcional cerebral. Para melhor compreender o declínio cognitivo na EM, esse artigo propões uma revisão dos principais métodos de imagem por RM e suas correlações com função cognitiva, discutindo as múltiplas faces patológicas da EM e seu impacto na eficiência das redes neurais.

## INTRODUCTION

Multiple sclerosis (MS) is a common inflammatory and demyelinating disease of the central nervous system (CNS). Although about 40 to 70% of MS patients develop significant cognitive decline,^1^^,^ 2 it was not until the past few decades that MS-related cognitive deficit is being systematically studied. Overt dementia in MS is rare, but the cognitive impairment can be substantially debilitating, impacting on patients daily activities.^3^

**Figure 1.**
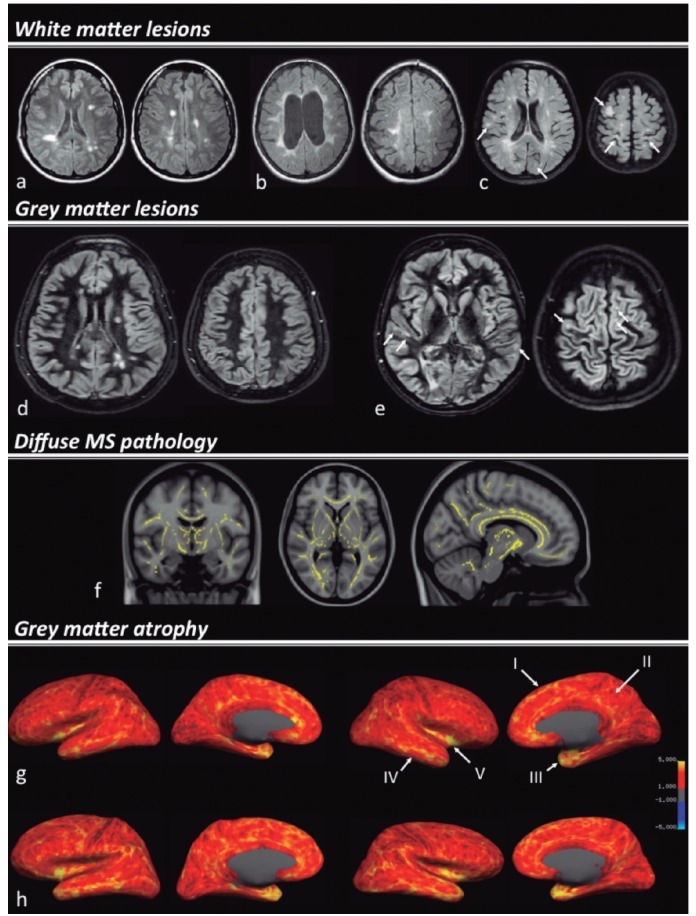
MRI metrics associated to brain damage and MS-related cognitive impairment. Examples of the multiple facets of MS pathology, white matter [A, B and C), grey matter lesions [D and E], diffuse MS pathology [F] and cortical grey matter atrophy [G and H] are demonstrated in the picture. White matter lesions are assorted in MS patients, with different distributions, according to the cognitive status. A cognitively preserved RRMS patient shows a lower lesion load, with predominance of centrally distributed WM lesions [A]; a SPMS patient [B] with mild cognitive impairment presents a higher lesion load, with confluent lesions, some of them reaching juxta-cortical locations; the more cognitively impaired subject [C], although this patient presents a lower overall lesion load than patient "b", the image shows an increased rate of (juxta)cortical lesions. Double inversion recovery (DIR) images [D and E] are sensible to detect cortical lesions. A cognitively impaired MS patient [E] has a significant higher number of cortical lesions (white arrows) than a cognitively preserved patient [D]. Fractional anisotropy differences between cognitively impaired and cognitively preserved MS patients [F - reproduced from Hulst et al.^77^, with permission of Wolters Kluwer). The brain areas in yellow display reduced fractional anisotropy in cognitively impaired patients compared to cognitively preserved patients. Cortical thickness maps in a cognitively impaired MS patient [G] and a healthy control [H]. The brain images in the left represent the measurements in the lateral and medial surfaces of the left hemisphere; the brain images in the right represent the surfaces of the right hemisphere (the cortical thickness is represented in a red to yellow scale). The MS patient shows reduced cortical thickness in several associative cortical areas in both hemispheres, compared to the healthy subject, remarkably in the superior frontal gyrus (I), posterior cingulate (II), temporal pole (III), temporal lobe (IV) and insula (V).

Although MS has been traditionally classified as a white matter (WM) disease, involvement of grey matter (GM) by demyelination and neurodegeneration has become evident in all stages of the disease.^4^^-^ 6 Neurodegenerative GM processes, culminating in axonal and neuronal injury, are being recognized as more direct causes of clinical disability and cognitive impairment.^4^^,^ 7 However, *in vivo* characterization of GM pathology remains challenging. Detection of focal GM lesions, especially in the cortical GM, requires advanced magnetic resonance imaging (MRI) techniques, such as high-resolution 3D-T1 and 3D-FLAIR,^8^ double inversion recovery (DIR)^9^ (Figure 1) and (ultra) high-field/ultra high-field scanners.^10^ Furthermore, it has been suggested that other factors, such as diffuse pathology in normal appearing brain tissues (NABT) are significant determinants of GM degeneration and atrophy,^11^^,^ 12 partly due to axonal transection in MS lesions^13^ are significant determinants of GM degeneration and atrophy.

It has been suggested that WM lesions and inflammatory processes have stronger influence as determinants of GM damage in early stages of MS,^14^^-^ 16 while neurodegenerative aspects and diffuse pathology in normal appearing WM (NAWM) and GM (NAGM)^12^^,^ 14 ^,^ 17 are more important determinants of brain damage in long-standing disease. However, a recent longitudinal study has shown that inflammatory changes and T2 lesion volumes remained predictive of disability even after 20 years in patients who presented with clinical isolated syndromes (CIS).^18^ Other studies have also demonstrated that different patterns of lesion distribution correlate to regional GM atrophy^13^ and rate of disease progression^19^ in primary progressive (PPMS) and secondary progressive (SPMS) MS. 

MRI is the main modality used to assess pathological changes in MS and it has been used to quantify WM lesions,^15^^,^ 20 ^,^ 21 GM lesions,^8^^,^ 10 ^,^ 22 diffuse abnormalities^11^^,^ 12 ^,^ 15 ^,^ 23 ^,^ 24 and atrophy^24^^-^ 26 (Figure 1). All these factors are present from early stages of MS,^16^^,^ 27 ^-^ 29 associate with higher disability scores and cognitive decline,^11^^,^ 15 ^,^ 16 ^,^ 30 and are more pronounced in long-standing disease.^14^^,^ 31 A few multi-parametric studies assessed several MRI measurements concurrently and have shown that MRI measures of MS pathology are highly interdependent and most of them will eventually correlate with disease severity.^12^^,^ 14 ^,^ 32

The neurodegenerative process in MS is likely to precipitate changes in brain function. Conventional MRI data (i.e. lesion volume, lesion location and atrophy) has been compared to neuropsychological tests scores to relate MS pathology to cognitive performance.^20^^,^ 21 ^,^ 25 ^,^ 33 However, this type of investigation provides indirect associations between structural damage and brain function. In contrast to conventional MRI, functional MRI (fMRI) is providing more direct visualization of brain activity. Recent studies have observed different synchronization patterns of neuronal activity that can be described as large-scale networks.^34^^-^ 36 These networks appear to be closely related to complex brain functions and can be used to study the organization of cognitive functions.^34^ Neuronal activity in individual components of functional networks is presumably integrated through WM pathways, building up structural modules of brain networks.^37^ Understanding the structural basis of functional connectivity requires comprehensive maps of structural connections of human brain. Recent advances in diffusion tensor imaging (DTI) and tractography are providing insights about WM microstructure, allowing noninvasive mapping of inter-cortical and deep GM anatomic connections.^37^^,^ 38 Recent assessments of brain networks in MS patients show that changes in cognitive performance are accompanied by variable modifications in functional^39^^,^ 40 and structural^38^^,^ 41 connectivity. These emerging imaging processing techniques are promising tools to surmount the limitations of conventional MRI in providing information about the integration of brain regions and systems. 

This paper reviews the associations between MRI metrics and cognitive performance in MS, by discussing the interactions between multiple facets of MS pathology as determinants of brain damage. We also present an overview of new imaging analysis techniques studying structural and functional brain networks towards accessing the interplay between structural damage, functional changes and cognitive outcome. 

## COGNITIVE DYSFUNCTION IN MS

Cognitive dysfunction in MS is heterogeneous and usually affects multiple cognitive domains. The more frequently affected domains are memory, attention, information processing speed and executive functions.^25^^,^ 32 Essential verbal skills (word naming and comprehension) remain relatively preserved, and it is rare to observe aphasia.^1^ A recent study showed that the spectrum of affected domains is relatively constant throughout the course of the disease: in long-standing MS patients the same cognitive domains were affected as in early MS, although the changes were more pronounced.^32^

Risk factors of cognitive symptoms in MS are not completely understood, but studies have suggested that sex, age and genetic predisposition may play a role. For instance, several studies reported that cognitive symptoms are more severe and occur more often in male patients than in female patients.^25^^,^ 42 Moreover, in adult patients with MS, cognitive symptoms seem to be worse with older age of onset.^43^ Lastly, the apolipoprotein E4 allele (APOE4), a gene associated to the sporadic form of AD, was reported as potential risk factor for more rapid disease progression and cognitive decline in MS, whereas the more common susceptibility genes for MS, such as human leukocyte antigen class II (HLA-II), do not specifically relate to cognition.^44^

Neuropsychiatric factors are an important aspect to consider when assessing cognitive performance in patients with MS: studies have shown that cognitive fatigue might be partially responsible for decreases in performance of tasks that require sustained mental effort.^45^ Depression is also common in MS, being present in up to 60% of the patients, and may impair the performance in working memory, processing speed, learning, abstract reasoning and executive functioning.^1^

Not surprisingly, progressive MS phenotypes present worse cognitive performance than early relapsing remitting MS (RRMS) or CIS patients.^1^ Some studies^32^^,^ 46 found poorer cognitive performance in both PPMS and SPMS compared to RRMS, though SPMS patients had a worse performance than PPMS when tasks required higher-order working memory processes.^46^

Although cognitive deficits can be found in early stages of the disease, they are usually a feature of advanced disease. Natural history studies suggest that the cognitive decline tends to progress with increased disease duration and, once it appears in MS patients, it is unlikely to remit.^47^^,^ 48 Hence, it seems that the cognitive deterioration in MS is a neglected aspect of disease progression.

## MRI CORRELATES OF COGNITIVE DYSFUNCTION

**White matter (WM) lesions.** Depicting WM lesions disseminated in the CNS is indispensible for the diagnosis and early clinical management of MS.^49^ Although some studies have reported associations between lesion volumes and cognitive impairment in specific cognitive domains, such as information and processing speed, verbal memory,^16^^,^ 50 sustained attention and executive functions,^16^ overall WM lesion load has shown only poor correlations with average cognition^50^ and progression of cognitive impairment.^32^^,^ 51 These poor correlations in part reflect the lack of pathological specificity of conventional MRI techniques^52^ and underestimation of the amount of demyelination and degeneration in NABT.^11^^,^ 17

It has been suggested that lesion location might be a stronger determinant of neurological dysfunction in MS,^20^^,^ 21 ^,^ 53 rather than overall lesion burden. Some studies have found significant correlations between periventricular lesion burden and physical disability.^53^^,^ 54 However, presence of lesions in more peripheral locations has shown stronger associations to cognitive decline (Figure 1C); a higher number and volume of juxta-cortical lesions has been observed in cognitively impaired patients,^21^^,^ 33 ^,^ 55 ^,^ 56 demonstrating significant correlations with average cognition,^21^^,^ 33 executive function and memory.^55^ Furthermore, specific correlations with regional lesion load have been reported, e.g. for verbal memory with lesions in temporal^20^ and parietal lobes;^57^ sustained attention, information and processing speed and executive function with lesions in frontal^20^^,^ 55 ^,^ 57 ^,^ 58 and temporal lobes;^20^ abstract reasoning with frontal lobe lesions^57^ and visuospatial memory with parietal^20^^,^ 57 and temporal^20^ lesions. It thus seems that periventricular and deep WM lesions might play a more important role in causing physical disability,^54^ while juxta-cortical lesions and lesions affecting associative pathways have greater impact on neuropsychological functioning.^20^^,^ 21

Most studies evaluating associations between lesion load and cognition were performed in patients with relative short disease duration. Baseline T2-hyperintense and T1-hypointense lesion loads are significant short-term predictors of disability and cognitive decline in patients with clinical isolated syndromes (CIS)^15^ and relatively short-term follow-up of RRMS^16^^,^ 59 and PPMS^19^. Increases in lesion volumes show greater impact on clinical outcome in the first 5 to 10 years of MS,^18^^,^ 19 with decreasing importance in the subsequent years suggesting that mechanisms of brain damage may change over time, and other factors such as GM lesions, diffuse MS-pathology and brain atrophy may be more important determinants of cognitive dysfunction in later stages. 

**Gray matter (GM) lesions.** Renewed interest in MS GM pathology has revealed that the prevalence of GM lesions is high^4^^,^ 60 and can affect cortical GM^60^ and also deep GM structures, such as thalamus, basal ganglia^61^ and hippocampus.^62^ Furthermore, it has been shown that abnormalities in and around the cortex are related to both physical and cognitive deficits.^10^^,^ 21 However, it is also known that more routinely used MRI sequences to investigate MS lesions, such as FLAIR and dual-echo T2-weighted images, fail to detect a large number of GM lesions.^60^

Double inversion-recovery (DIR) is a relatively new MRI sequence that selectively nulls the signals from WM and cerebrospinal fluid (CSF), leaving only GM and lesions visible^62^^,^ 63 (Figure 1D and 1E). When applied to a MS population it resulted in a 5-fold increase in detection rate of cortical lesions compared to standard MRI pulse-sequences.^60^. DIR also improved lesion detection in other GM regions, such as hippocampus^62^ and cerebellar GM.^64^ Cognitively impaired MS patients generally present higher cortical lesion number and volume than their cognitively unimpaired counterparts.^65^ A recent study has demonstrated that the GM lesions detected by DIR frequently affect cortical regions involved in information processing,^66^ being mainly distributed in frontal and temporal lobes, with prominent involvement of motor and anterior cingulate cortices. Moreover, GM lesions in specific associative cortices and hippocampal lesions are strongly associated with impaired visuospatial memory and information processing speed.^62^^,^ 67

Although DIR has considerably improved GM lesion detection, a post-mortem evaluation uncovered that about 80% of cortical lesions remain undetected at 1.5T.^68^ Especially purely intra-cortical and subpial lesions are still frequently missed. Several new strategies are being pursued to increase the detection of cortical lesions in MS. These include the combination of DIR with other MRI sequences, such as phase-sensitive inversion recovery (PSIR),^69^ magnetization-prepared rapid acquisition gradient echo (MPRAGE),^10^ or the use of ultra-high field scanners.^10^ A recent study found an improved rate of cortical lesion detection using MPRAGE using a 7T scanner, observing stronger correlations between the number of cortical lesions and cognitive tests and EDSS scores.^10^ Additionally, the use of ultra-high field scanners facilitates the characterization of cortical lesion subtypes, especially improving *in vivo* visualization of the most prevalent subtype - subpial lesions.^70^ The latter study showed that, within the cortical lesion subtypes, mixed grey-white matter lesions and subpial lesions have the strongest correlations with cognitive and physical disability status in MS.

Despite the fact that multiple studies have shown the clinical relevance of cortical lesions, there are still several limitations to the assessment of cortical lesions in routine clinical practice. First, specific MRI sequences to detect cortical lesions, such as DIR and PSIR, are not available on most clinical scanners. The availability of ultra-high field scanners for clinical practice is even more limited. Second, DIR imaging is prone to artifacts^62^ and still has low sensitivity to detect subpial lesions.^68^ Third, the inter-observer agreement for the assessment of cortical lesions on DIR sequences is sub-optimal^71^ and assessors need to be trained to read these images. Finally, the majority of cortical thinning occurs independently of cortical lesions, suggesting that there are mechanisms contributing to GM degeneration beyond focal demyelination.^72^

**Diffuse MS pathology.** Quantitative MRI techniques, such as magnetization transfer imaging (MTI) and diffusion tensor imaging (DTI), enable quantification of the extent and severity of structural changes occurring in NABT outside focal lesions.^52^ MTI quantifies the macromolecular content in brain tissues through the transfer of magnetization between immobile (protein-bound) and free (water) protons.^52^^,^ 73 Loss of myelin and axonal membranes in MS lesions or NABT reduces the pool of protons bound to macromolecules and increases the mobile proton pool, decreasing the magnetization transfer ratio (MTR).^73^ The most commonly used DTI indexes to assess the microstructural characteristics of brain tissues include fractional anisotropy (FA) and mean diffusivity (MD).^12^^,^ 74 Extra-cellular edema, myelin and axonal membrane disruption deviate the water diffusion from a highly oriented (i.e. anisotropic) to a more isotropic condition, decreasing FA and increasing MD.^12^

Several studies have shown that correlations between cognitive deterioration in MS and markers of diffuse MS pathology are stronger than correlations observed between cognitive decline and WM lesion load.^16^^,^ 32 ^,^ 42 ^,^ 75 ^,^ 76 Cognitively impaired MS patients present lower average MTR and peak location in (NABT) histograms^11^ and greater extent and severity of DTI parameters abnormalities.^32^^,^ 42 ^,^ 77 A longitudinal study showed that changes in NABT MTR are stronger predictors of cognitive outcome in MS patients than increases in lesion volumes.^16^ Furthermore, a recent study was able to distinguish cognitively preserved from cognitively impaired MS patients, based on characteristics of microstructural WM damage measured with DTI.^77^ MS patients showed reduced FA in several brain areas, being significantly worse in cognitively impaired subjects (Figure 1F). In addition, some areas critical for cognition, such as the thalamus, uncinate fasciculus and cerebellum were only affected in the cognitively impaired group. Aligned to these observations, another recent multi-parametric study found that NAWM FA and deep GM atrophy were the strongest predictors of cognitive dysfunction in long-standing MS.^32^

Evidence suggests that MTR and DTI can be used to probe neurodegeneration (i.e. indirect signs of neuronal loss) and might have prognostic value. First, MTR^23^ and DTI^12^^,^ 14 abnormalities worsen along the course of MS, being more pronounced in progressive subtypes. Second, DTI changes in NABT are closely related to GM atrophy.^14^^,^ 78 Finally, changes in MTR NAWM progress in SPMS partially independent from the formation of new MS lesions^79^ and were considered to be resistant to first-line immunomodulatory drugs, such as interferon beta-1.^80^

However, most of the MRI parameters used to investigate NAWM and NAGM abnormalities (i.e. MTR, FA and MD) are pathologically unspecific, and might correspond to (combinations of) demyelination, inflammatory processes or neurodegeneration.^79^ Furthermore, the observed abnormalities are quantitatively small and there are no current standardized parameters to be applied on an individual level.^79^

**Brain atrophy.** Brain atrophy is considered to be an end-stage phenomenon, strongly correlated to permanent brain damage / neurodegeneration. Patterns of GM atrophy are being identified from early stages of MS onward^81^ and show differential effects on neuropsychological functioning. Deep GM atrophy is more pronounced than cortical atrophy in the initial stages of the disease and in patients with clinical isolated syndromes (CIS).^5^^,^ 82 Mesial temporal (i.e. hippocampal and amygdala)^83^^,^ 84 and thalamic^25^^,^ 78 ^,^ 85 atrophy are the most frequent deep GM structures associated with cognitive impairment in MS. Thalamic atrophy seems to affect multiple cognitive domains, such as executive functions, information and processing speed, attention and psychomotor processing speed,^25^ while the hippocampal damage is more specifically associated with memory impairment.^82^ Thalamus and caudate atrophy also play a role in memory impairment, but with different manifestations than found for mesial temporal structures. While thalamus and caudate atrophy were the primary predictors of impaired learning and new memory acquisition (i.e. memory encoding), mesial temporal atrophy showed stronger correlations with recognition of recently learned information (i.e. memory retrieval).^86^

Global cortical thinning is mild in early stages of MS such as CIS and increases with disease severity^27^^,^ 87 and in conjunction with cognitive decline^65^ (Figure 1G). There are some patterns of regional cortical atrophy in MS that correlate with disability,^88^ specific clinical symptoms^27^^,^ 89 and cognitive decline.^90^ Calabrese et al.^27^ found significant cortical atrophy in frontal, precentral and occipital areas in very early stages of MS and the degree and distribution of cortical thinning was associated with symptoms at disease onset. In another study, atrophy in the striatum, posterior parietal cortex and middle frontal gyrus correlated with fatigue.^89^ Charil et al.^88^ observed correlations between EDSS and cortical thickness in the anterior cingulate gyri, insula and frontal and temporal associative cortices. One study showed that GM volumes in bilateral prefrontal cortex, precentral gyrus, superior parietal lobe, left precuneus and right cerebellum correlate with Paced Auditory Serial Addition Task (PASAT),^91^ a neuropsychological test used to assess information processing speed and sustained attention. A recent study detected non-random patterns of cortical atrophy in MS, demonstrating that the spatial distribution of cortical atrophy is closely related to the dominant clinical profile.^90^ Interestingly, the patterns that showed most pronounced cortical atrophy overlap with regions associated to the limbic system and the default mode network (DMN), and atrophy in the posterior cingulate cortex and temporal pole was associated to cognitive dysfunction.

Despite these efforts, it is still not well understood which pathological factors determine GM atrophy in MS. Previous studies showed associations between GM atrophy with WM,^87^^,^ 88 GM^72^ lesions and NAWM damage.^14^^,^ 29 Most of these studies were restricted to a single MRI modality or brain region, and investigated patients with a relatively short disease duration, which makes it hard to discern the importance of individual measurements. A recent multi-modality study showed that all these factors play independent roles as determinants of GM atrophy.^14^ Lesion load and diffuse WM pathology were important explanatory variables for cortical thickness and deep GM atrophy in RRMS. On the other hand, WM atrophy and changes in NAWM (on DTI) were stronger predictors of GM atrophy in progressive types of MS. Although the pathological mechanisms in MS might change over time, it seems that GM degeneration occurs at least partly secondary to WM damage. The complex substrate of brain damage and cognitive dysfunction in MS emphasizes the need for more comprehensive approaches, investigating the interplay of different imaging markers, towards assessing the links between structural damage and functional changes.

## BRAIN CONNECTIONS AND NETWORKS: THE MISSING LINKS BETWEEN STRUCTURAL DAMAGE AND FUNCTIONAL OUTCOME

**Figure 2.**
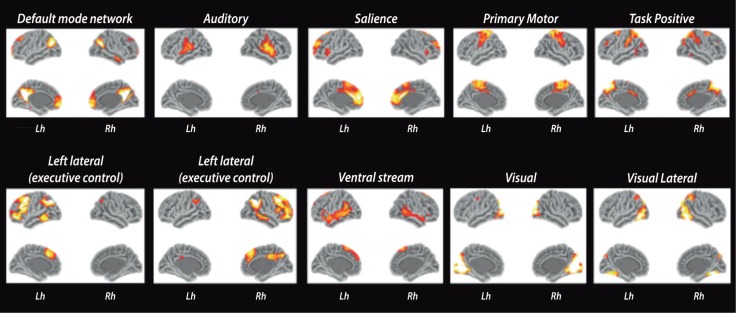
Resting-state fMRI networks. The ten main components of resting-state networks. Images were obtained after processing functional images of 30 healthy controls using FSL toolbox.

**Figure 3.**
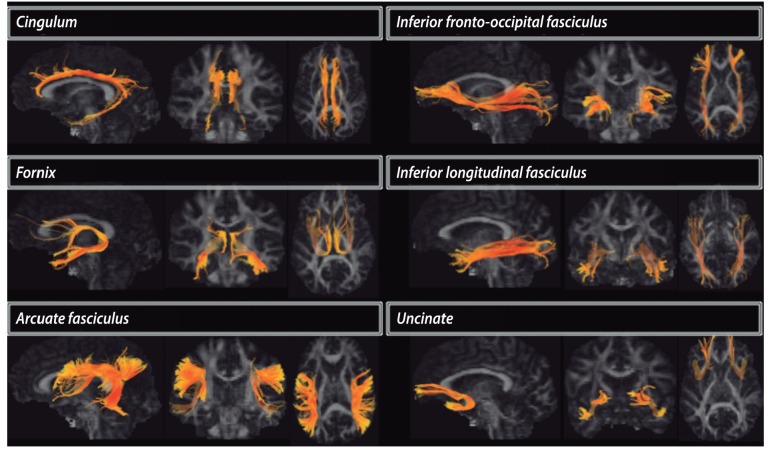
Reconstruction of WM tracts using DTI tractography. The cingulum is one of the most important components of the limbic system, and it has been associated with attention, temporal orientation, information processing and executive functions. The fornix also integrates the limbic system, connecting the hippocampus and amygdala to the mammillary bodies and then to prefrontal regions through the anterior forebrain and thalamus. The arcuate fasciculus connects the posterior temporal and inferior parietal cortices to the frontal lobe, and it has been linked to primary and executive language functions and verbal memory. The inferior fronto-occipital fasciculus and the inferior longitudinal fasciculus have a partial spatial overlap, connecting the occipital cortex to the fronto-orbital and anterior temporal regions. They have been associated with visual memory, visuospatial functions and object recognition. The uncinate fascicullus connects the temporal pole to fronto-orbital regions, being associated with memory, learning and information encoding.

Traditional models of brain function employ modular paradigms, in which brain areas are postulated to act as independent processors for specific neural functions.^34^ Accumulating evidence suggests that segregation of brain areas in highly specialized modules is overly simplistic and presents several limitations in explaining higher cognitive functions. Newer paradigms in cognitive neuroscience show evidence of complex cross-modal interactions where conjoint functions of brain areas work together in large-scale networks.^34^ A more comprehensive brain model can be summarized as multiple conjuncts of GM areas connected by WM pathways or networks. Using MRI, the functional connectivity is usually assessed by resting-state functional MRI (fMRI)^35^^,^ 40 ^,^ 92 (Figure 2), while structural WM pathways are being accessed by DTI tractography^38^^,^ 93 ^-^ 95 (Figure 3). 

Most DTI studies show signs of microscopic damage/disconnection in several WM pathways involved in cognition. The fiber bundles more frequently associated with neuropsychological performance are the cingulum,^93^^,^ 94 uncinate fasciculus (UF),^93^^,^ 95 corpus callosum,^30^ superior and medial cerebellar peduncles.^93^ The damage in these fiber bundles is associated with interlobar, interhemispheric and long-distance brain disconnection. In addition to the disconnection of long-distance WM pathways, two studies observed signs of decreased local efficiency in prefrontal and occipital associative areas.^38^^,^ 94

Some of the disrupted WM connections are anatomically related to the default mode network (DMN).^38^^,^ 94 The DMN is one of the most exuberant resting-state functional networks and appears to be relevant in maintaining normal cognition and higher executive functions. It concatenates parietal, occipital and prefrontal cortical functions,^96^ with important participation of precuneus and cingulate cortex. Some fMRI studies showed decreased connectivity in the DMN^35^^,^ 36 ^,^ 96 ^,^ 97 in MS, which are related to cognitive impairment. Other studies found increased functional activity/connectivity in components of the DMN^92^^,^ 98 and different cognitive networks, such as attention,^97^ hippocampal^98^ executive and auditory.^36^ Increased functional activity was most often reported in cognitively preserved MS patients. However, studies reported positive,^97^^,^ 99 negative^36^ or both positive and negative^98^ correlations between increased connectivity and cognitive performance or disability level.

These results are difficult to interpret. Increased connectivity in cognitively preserved patients could be a sign of beneficial functional reorganization or compensatory mechanisms to sustain normal function, delaying the cognitive decline. Alternatively, it could be a maladaptive response secondary to the disruption of inhibitory WM connections. In this context, one would expect positive correlations between signs of WM disconnection and increased functional connectivity. Louapre et al.^97^ observed significant correlations between increased functional activity in the attention network and DTI abnormalities in anterior and posterior cingulum. A positive correlation between WM microstructural damage and increased functional activity was also observed in hippocampal pathways.^41^ On the other hand, a recent multimodality study^100^ showed results in the opposite direction, reporting diffuse WM microstructural damage being associated with decreased functional connectivity in 5 resting-state networks, with both positive and negative correlations between WM damage and functional activity. 

Different studies found similar results in structural^38^^,^ 94 and functional^40^^,^ 92 networks in thalamic and cingulate connectivity. They showed that, in mildly impaired patients, both functional and structural connectivity are increased. However, there are no studies that systematically investigated global structural and functional connectivity in the same patients. Also, most of the studies are cross-sectional, thus it cannot be excluded that different results between studies are secondary to cognitive reserve and inter-subject differences. 

## CONCLUSION

Cognitive decline is an important source of disability in MS patients and an undervalued aspect of disease progression. Even though cognitive impairment is partly related to macroscopic lesion load, focal damage might play an indirect role as a cause of further tissue damage. Reflective of permanent damage and tissue loss, atrophy markers show stronger correlations with cognitive tests, but regional damage in target structures provides limited information about their specific relevance for a given cognitive domain and the integration between brain areas and systems. A more holistic investigation of brain networks seems to better capture the mechanisms of brain damage and (mal)adaptation encountered in cognitive dysfunction. However, multimodal imaging techniques and network analyses are still in their infancy and, although it is possible to visualize the associative areas more frequently affected in cognitive impaired MS subjects, we still lack a more specific comprehension of the interplay between function and structure to better understand initial adaptation and subsequent collapse of brain networks. 
